# Clinical analysis of pregnancy complicated with miliary tuberculosis

**DOI:** 10.1080/07853890.2021.2018485

**Published:** 2021-12-27

**Authors:** Kaige Wang, Donghua Ren, Zhixin Qiu, Weimin Li

**Affiliations:** a Department of Pulmonary and Critical Care Medicine, West China Hospital, Sichuan University, Chengdu, China; b Department of Pulmonary and Critical Care Medicine, Xining Second People's Hospital, Xining, China

**Keywords:** Pregnancy, IVF-ET, miliary tuberculosis, bone marrow tuberculosis, ARDS

## Abstract

**Background:**

Pregnancy complicated with tuberculosis is increasingly common. The clinical characteristics of pregnancy complicated with miliary tuberculosis are summarized in this study.

**Methods:**

A retrospective analysis of pregnant patients with miliary tuberculosis was performed in terms of epidemiology, demography, clinical characteristics, laboratory tests, treatment, and prognosis.

**Results:**

Of the 23 patients that were included, 12 became pregnant after *in vitro* fertilization combined with embryo transfer (IVF-ET). The average gestational age at symptom onset was 13.96 weeks, and the average time from symptom onset to diagnosis was 33 days. Clinical symptoms included fever, dyspnoea, cough, headache, abdominal pain, and chest pain. Extrapulmonary tuberculosis occurred in 10 patients, respiratory failure in 11 patients, and ARDS in 9 patients. Chest HRCT showed diffusely distributed miliary nodules in all patients. Six patients were on mechanical ventilation, two underwent ECMO, and one died. Symptoms appeared in the first trimester of nine pregnancies after IVF-ET and in the second trimester of seven natural pregnancies.

**Conclusions:**

Miliary tuberculosis can occur in pregnant patients, especially in patients after IVF-ET. Symptoms often appear in the first trimester of pregnancy after IVF-ET and in the second trimester of natural pregnancy. Lacking specificity, the common clinical characteristics include elevated inflammation markers, anaemia, low lymphocyte count, and multiple miliary nodules shown on a chest HRCT scan. Half of patients with miliary tuberculosis may develop respiratory failure, and some may progress to ARDS. Therefore, infertile patients should be required to undergo TB screening before undergoing IVF-ET, and preventive anti-TB treatment should be given to patients with latent TB infections or untreated TB disease.

**Key Message**
- Miliary tuberculosis can occur in pregnant patients, especially in pregnant patients after IVF-ET. Symptoms often appear in the first trimester of pregnancy after IVF-ET and in the second trimester of natural pregnancy. Many patients develop respiratory failure or ARDS.

## Background

The global burden of tuberculosis (TB) remains enormous [1], especially in developing countries [2]. TB is a major cause of poor health, 1 of the top 10 causes of death worldwide, and the leading cause of death from a single infectious agent. In 2019, an estimated 10 million people were newly infected with TB worldwide, along with an estimated 1.2 million TB deaths among HIV-negative people. TB affects people of both sexes in all age groups, in which women account for 32% [3]. Maternal TB is associated with poor prognosis in both the mother and foetus and may lead to infant and maternal mortalities. As early as 1984, a case of miliary TB in an *in vitro* fertilization (IVF) pregnancy was reported by Gail M. for the first time [4]. With technical promotion, *in vitro* fertilization combined with embryo transfer (IVF-ET) has become a widely used infertility treatment worldwide, resulting in an increasing number of pregnant patients with miliary TB after IVF-ET. However, there have been few reports on this disease thus far [5,6]. In this study, a clinical analysis of 23 cases of pregnancy complicated with miliary TB was performed in terms of clinical symptoms and laboratory test results, along with a comparative analysis of the clinical data between the IVF-ET and non-IVF-ET groups.

## Methods

### Study design

A retrospective analysis of pregnant patients with miliary TB admitted to West China Hospital of Sichuan University from July 2015 to June 2020 was performed in terms of clinical characteristics and laboratory test results. Ethics approval was granted by the Ethics Board of the Institute of West China Hospital of Sichuan University (2013-131). Written informed consent for publication was obtained only for case report.

### Data analysis

Data on epidemiology, demography, clinical characteristics, laboratory tests, treatment and prognosis were obtained from the electronic medical records of West China Hospital. All the data were reviewed by two infectious disease doctors, and then a third researcher (L. D.) determined whether there was any difference in interpretation between the two reviewers.

### Laboratory tests

All patients admitted to our hospital through the outpatient or emergency department underwent the following: sputum smear microscopy (acid-fast staining ≥3 times), sputum-based TB-DNA detection (2 times), sputum-based TB culture (≥1 time), electrocardiogram (ECG), arterial blood gas analysis, complete blood count (CBC) tests, coagulation function tests, serum biochemical analyses (including liver and kidney function, creatine kinase levels and lactate dehydrogenase levels), cardiac enzyme level tests, brain natriuretic peptide (BNP) level tests, C-reactive protein (CRP) level tests, erythrocyte sedimentation rate (ESR), procalcitonin (PCT) level tests, purified protein derivative (PPD) skin test, and TB-interferon gamma release assay (TB-IGRA). At least one chest CT scan was performed for each patient (the exam frequency was subject to a doctor’s orders). Some patients also underwent bone marrow puncture, thoracic puncture, abdominal puncture, or lumbar puncture.

### Definition

Pregnancy is divided into three stages: early pregnancy refers to the first trimester (weeks 1 through 13 of pregnancy); middle pregnancy refers to the second trimester (weeks 14 through 27 of pregnancy); and late pregnancy refers to the third trimester (28th week through the end of the pregnancy).

Miliary TB is the pathological name describing millet seed-sized (1–2 mm) granulomas in various organs affected by tubercle bacilli. It results from massive lymphohaematogenous dissemination from a *Mycobacterium tuberculosis*-laden focus. A miliary TB diagnosis was established based on the presence of clinical and radiological signs and confirmed by aetiological diagnosis, pathological diagnosis or therapeutic response [7]. The diagnosis of acute respiratory distress syndrome (ARDS) used the Berlin definition of ARDS [8].

### Statistical methods

The data were analyzed by means of SPSS version 20.0 (SPSS Inc., Chicago, IL). The normally distributed data are expressed as the mean ± SD. A *t* test was used to compare the means of the two groups, and variance analysis was used for comparison. The nonnormally distributed measurements were expressed as the median and analyzed by the Wilcoxon rank-sum test. The Chi-square test, denoted by *χ*^2^, was used to analyze enumeration data, and a *p* value of less than .05 was considered statistically significant.

## Results

### Clinical characteristics

There were 74 pregnant women with TB. Among them, 45 became pregnant naturally and 29 became pregnant after IVF-ET, of which 21 women had secondary pulmonary tuberculosis, 13 had extrapulmonary tuberculosis, and 17 had tuberculous pleurisy. Before embryo transfer, 8 patients received glucocorticoid treatment with 10 mg of prednisone per day for 15–30 days. None of the patients in this study had diabetes. A total of 23 pregnant women with miliary TB were included in this analysis, with an average age of 28.4 years (range: 20–44 years). Among them, 12 became pregnant after IVF-ET, and the other 11 became pregnant naturally. Thirteen patients reported having one or more abortions, of which 8 women had natural pregnancies and 4 had two or more abortions. No natural pregnancy was reported in the IVF-ET group, where miscarriage occurred in five patients after the onset of TB, and the rest underwent abortion induction or labour induction after diagnosis. Three patients in the IVF-ET group had twin pregnancies, while all patients in the natural pregnancy group had singleton pregnancies. One patient reported having tuberculous pleurisy, and another reported having transient pleural effusion; none of them had previously received any anti-TB therapy. The average gestational age at symptom onset was 13.96 weeks (range: 2–40 weeks). The time from symptom onset to diagnosis ranged from 7 to 120 days, with an average of 33 days. The average length of stay in our hospital was 23.96 days. The women’s clinical symptoms included fever, dyspnoea, cough, headache, abdominal pain, and chest pain. Extrapulmonary TB occurred in ten patients, of which 3 had tuberculous pleurisy, 3 had abdominal TB, 4 had tuberculous encephalopathy and 2 had bone marrow TB. Pneumothorax occurred in three patients. Eleven patients developed respiratory failure, and eight presented with ARDS (Table 1). Before embryo transfer, 8 patients received glucocorticoid treatment with 10 mg of prednisone per day for 15–30 days.

**Table 1.** Clinical characteristics of pregnancy complicated with military tuberculosis.

Characteristics	All	Natural pregnancies	Pregnancies after IVF-ET	*p* value
Numbers	23	11	12	
Average age, years, mean (SD)	28.39 (5.74)	27.45 (6.73)	29.25 (4.81)	.467
Pregnancy age at symptom onset, mean (SD)	13.96 (8.53)	16.82 (9.38)	11.33 (7.05)	.132
Cases with symptom onset in the first trimester (≤13 weeks)	12 (52.1)	3 (13.0)	9 (39.1)	**.022**
Cases with symptom onset in the second trimester (13–28 weeks)	10 (43.4)	7 (30.4)	3 (13.0)	.062
Twin pregnancy, *n* (%)	4 (17.4)	0	4 (33.3)	**.035**
Time from symptom onset to diagnosis, days, mean (SD)	33.00 (27.40)	38.45 (32.52)	28.00 (21.99)	.373
Length of stay in hospital, days, mean (SD)	23.96 (13.78)	25.82 (13.30)	22.25 (14.57)	.547
Initial symptoms, *n* (%)				
Fever	22 (95.7)	10 (90.9)	12 (100.0)	.965
Cough	12 (52.2)	6 (54.5)	6 (50.0)	1
Dyspnoea	16 (69.6)	9 (81.8)	7 (58.3)	.442
Chest pain	3 (13.0)	2 (18.2)	1 (8.3)	.936
Headache	4 (17.4)	2 (18.2)	2 (16.7)	1
Abdominal pain	4 (17.4)	2 (18.2)	2 (16.7)	1
Complications, *n* (%)				
Tuberculous pleurisy	3 (13.0)	2 (18.2)	1 (8.3)	.936
Abdominal tuberculosis	3 (13.0)	2 (18.2)	1 (8.3)	.936
Tuberculous encephalopathy	4 (14.7)	2 (18.2)	2 (16.7)	1
Bone marrow tuberculosis	2 (8.7)	2 (18.2)	0 (0.0)	.421
Pneumothorax	3 (13.0)	2 (18.2)	1 (8.3)	.936
Respiratory failure	11 (47.8)	6 (54.5)	5 (41.7)	.842
ARDS	8 (34.8)	5 (45.5)	3 (25.0)	.555
Concomitant bacterial infection	6 (26.1)	4 (36.4)	2 (16.7)	.549
Concomitant fungal infection	2 (8.7)	1 (9.1)	1 (8.3)	1
Treatments, *n* (%)				
Hormone use	11 (47.8)	8 (72.7)	3 (25.0)	.061
Mechanical ventilation	6 (26.1)	4 (36.4)	2 (16.7)	.549
Anti-TB regimens				
HREZ	13 (56.5)	NA	NA	NA
HL_2_EO	6 (26.1)	NA	NA	NA
HREO	1 (4.3)	NA	NA	NA
HEO	3 (13.0)	NA	NA	NA
Fate, died				
Pregnant woman	1 (4.3)	1 (9.1)	0	.965
Foetus	9 (39.1)	4 (36.4)	5 (41.7)	.833

### Laboratory tests

Laboratory tests and measurements included haemograms, serum biochemical analyses (including liver and kidney function, creatine kinase levels and lactate dehydrogenase levels), cardiac enzyme levels, coagulation function, d-dimer levels, BNP levels, CRP levels, ESR levels, PCT levels, T cell subsets levels, PPD skin test, TB antibody levels, TB aetiology and serum-based TB-IGRA (Table 2). Concomitant bacterial and fungal infections were found in six and two patients, respectively.

**Table 2.** Initial laboratory analysis of pregnancy complicated with military tuberculosis.

Initial laboratory analysis	*N* = 23, *n* (%), mean (SD)	Natural pregnancies	Pregnancies after VF-ET	*p* Value
Complete blood count				
White blood cell count; ×10^9^/L; normal range 3.5–9.5	7.17 (5.81)			
Increase	4 (17.4)	2 (18.2%)	2 (16.7%)	.138
Decrease	13 (56.5)	3 (27.3%)	0	.833
Lymphocyte count; ×10^9^/L; normal range > 1.1	0.73 (0.23)			
Decreased	21 (91.3)	10 (90.9%)	11 (91.7%)	.833
Neutrophil count; ×10^9^/L; normal range 1.8–6.3	5.41 (3.21)			
Increase	7 (30.4)	4 (36.4%)	3 (25.0%)	.825
Red blood cell count, ×10^9^/L; normal range 3.5–5	3.15 (1.30)			
Decrease	21 (91.3)	11 (100%)	10 (83.3%)	.366
Haemoglobin; g/L; normal range > 130	98.70 (14.87)			
Decrease	21 (91.3)	11 (100%)	10 (83.3%)	.499
Platelet count; ×10^9^/L; normal range 100–300	195.48 (108.31)			
Decrease	4 (17.4)	3 (27.3%)	1 (8.3%)	.459
reactive protein; mg/L; normal range < 10	66.8 (35.01)			
** **Increase	23 (100)	11 (100%)	12 (100%)	NA
Procalcitonin; ng/mL; normal range < 0.1	2.0 (4.28)			
Increase	23 (100)	11 (100%)	12 (100%)	NA
ESR; mm/h; normal range <15	55.04 (22.04)			
Increase	22 (95.7)	11 (100%)	11 (91.7%)	1
Cardial markers				
Creatine kinase-MB; ng/mL; normal range < 5	16.22 (14.18)			
** **Increase	13 (56.5)	23 (100%)	23 (100%)	NA
Troponin; ng/mL; normal range <0.04 ng/mL	13.33 (12.41)			
** **Increase	6 (26.1)	5 (45.5%)	1 (8.3%)	.121
Brain natriuretic peptide; pg/mL; normal range <900	583.78 (959.44)			
** **Increase	19 (82.6)	9 (81.8%)	10 (83.3%)	1
Blood biochemical analysis				
Total bilirubin	18.06 (15.58)			
** **Increase	2 (8.7)	2 (18.2%)	0	.152
Direct bilirubin	11.11 (14.42)			
** **Increase	8 (34.8)	5 (45.5%)	3 (25.0%)	.555
Alanine aminotransferase; U/L; normal range <50	61.87 (40.07)			
** **Increase	11 (47.8)	3 (27.3%)	8 (66.7%)	.141
Aspartate aminotransferase; U/L; normal range <40	75.48 (46.45)			
** **Increase	15 (65.2)	7 (63.6%)	8 (66.7%)	1
Creatinine; μmol/L; normal range < 111	51.43 (14.34)			
** **Increase	1 (4.3)	16 (31%)	3 (12%)	NA
Albumin; g/L; normal range >35	28.69 (4.09)			
** **Decrease	23 (100)	11 (100%)	12 (100%)	NA
Lactic dehydrogenase; U/L; normal range <250	406.04 (179.31)			
** **Increase	23 (100)	11 (100%)	12 (100%)	NA
Creatine kinase; U/L; normal range <310	62.09 (195.81)			
** **Increase	9 (39.1)	1 (9.1%)	0 (0%)	.833
Coagulation				
Prothrombin time; sec; normal range 9–16	12.03 (2.05)			
** **Increase	6 (26.1)	2 (18.2%)	4 (33.3%)	.725
Activated partial prothrombin time; sec; normal range 28–44	34.65 (9.14)			
** **Increase	6 (26.1)	2 (18.2%)	4 (33.3%)	.725
D-Dimer; mg/L; normal range <0.55	3.83 (3.08)			
** **Increase	59 (76.6)	11 (100%)	12 (100%)	NA
Cell immunity				
CD4+ cell count; /µL; normal range >404	367.61 (128.63)			
** **< 404/µL	10 (43.5)	4 (36.4%)	6 (50.0%)	.812
CD8+ cell count, /µL; normal range >220/µL	296.09 (116.13)			
** **Decrease	19 (82.6)	8 (72.7%)	11 (91.7%)	.518
Lactic acid in blood gas analysis; mmol/L; normal range <1.5	1.99 (1.41)			
Increase	9 (39.1)	5 (45.5%)	4 (33.3%)	.867
BCG skin test positive	12 (52.2)	6 (54.5%)	6 (50.0%)	1
TB-IGRA positive	15 (65.2)	7 (63.6%)	8 (66.7%)	1
Tuberculosis antibody positive	9 (39.1)	4 (36.4%)	5 (41.7%)	1
Sputum AFB positive	1 (4.3)	0	1 (8.3%)	1
Sputum TB-DNA positive	5 (21.7)	3 (27.3%)	2 (16.7%)	.912
Sputum TB-bacillus culture positive	3 (13.0)	1 (9.1%)	2 (16.7%)	1

### Imaging examinations

Diffusely distributed miliary nodules were observed on chest high-resolution computed tomography (HRCT) scans of all patients. Among them, 22 patients were diagnosed with acute miliary TB, characterized by evenly distributed nodules, 1–2 mm in diameter, and one patient was diagnosed with chronic miliary TB, characterized by unevenly distributed nodules, 2–4 mm in diameter, mainly in the upper and middle lung lobes. Clustered and partially consolidated nodules were observed in both lower lobes on the chest CT scans of eight patients with ARDS. Cord-like shadows and nodule shadows were seen at the apexes of both upper lobes in three patients, in line with the imaging findings of obsolete pulmonary TB.

### Treatment and prognosis

First-line anti-tuberculosis therapy was given to all the patients, of whom 11 received additional hormone therapy, 4 were on invasive ventilators, 2 were on non-invasive ventilators, and 2 underwent extracorporeal membrane oxygenation (ECMO). Twenty-two patients were relieved and discharged from our hospital after treatment, and one died of intracranial haemorrhage during ECMO treatment (Table 1). Among the 23 patients, 9 patients had spontaneous abortions before hospitalization, and 14 patients had labour induced to terminate their pregnancy after anti-tuberculosis treatment.

### Grouping analysis

Depending on their pregnancy status, all the patients included in this study were divided into two groups: the IVF-ET group and non-IVF-ET (natural pregnancy) group. The twin pregnancy rate in the IVF-ET group was significantly higher than that in the non-IVF-ET group. The IVF-ET group was more likely to experience early symptom onset. Symptoms appeared in the first trimester of nine pregnancies after IVF-ET and in the second trimester of seven natural pregnancies. No other difference was found in the clinical characteristics, laboratory test results, treatment or prognosis between these groups (Tables 1 and 2).

## Case presentation

**Figure 1.**
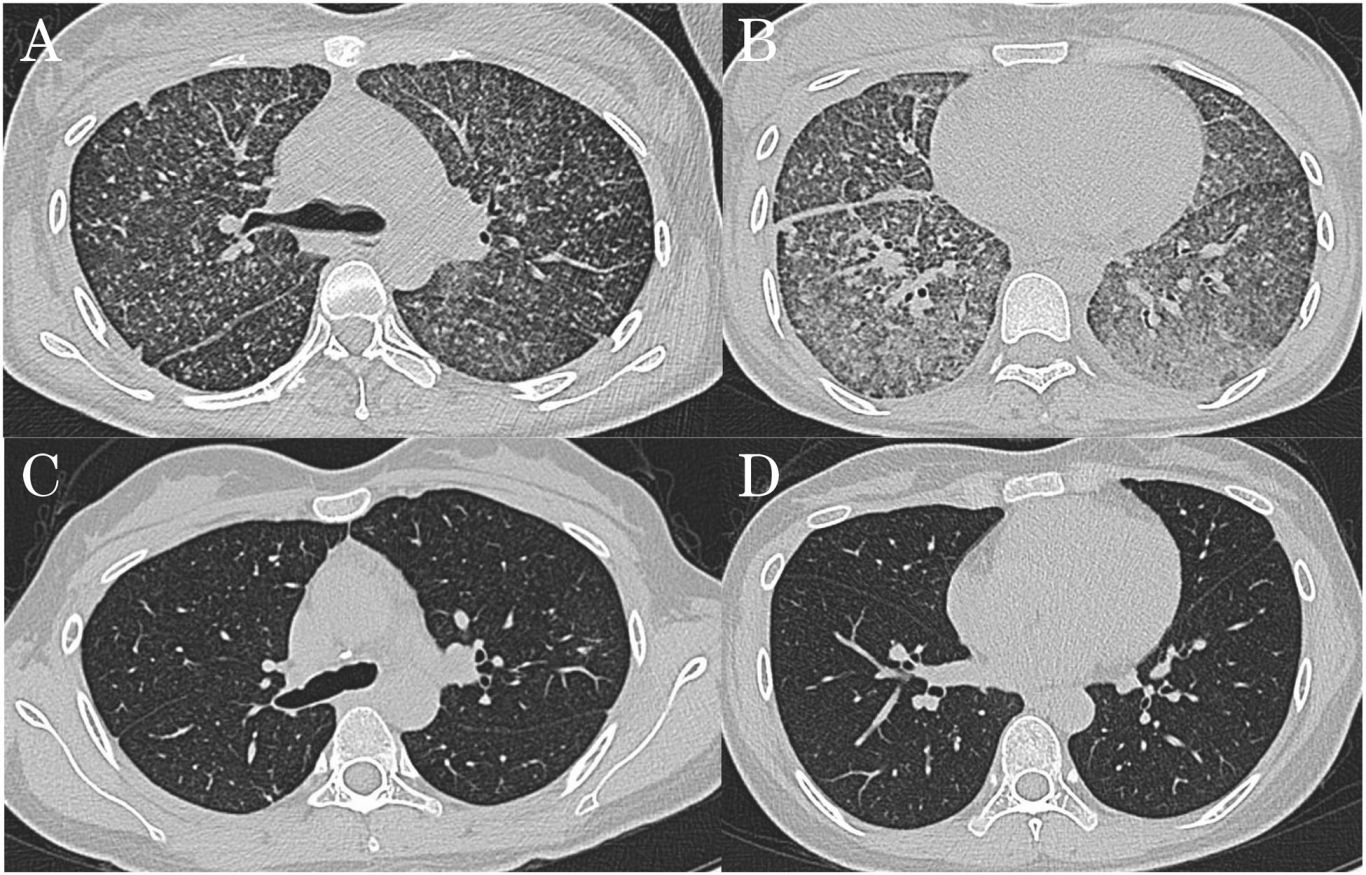
Image of chest CT scan. (A and B) Chest HRCT of a case on admission showed multiple miliary nodular lesions distributed diffusively in both lungs, partially clustered and consolidated. (C and D) Chest HRCT after 6 months treatment showed absorption of miliary nodular lesions in bilateral lung.

**Figure 2.**
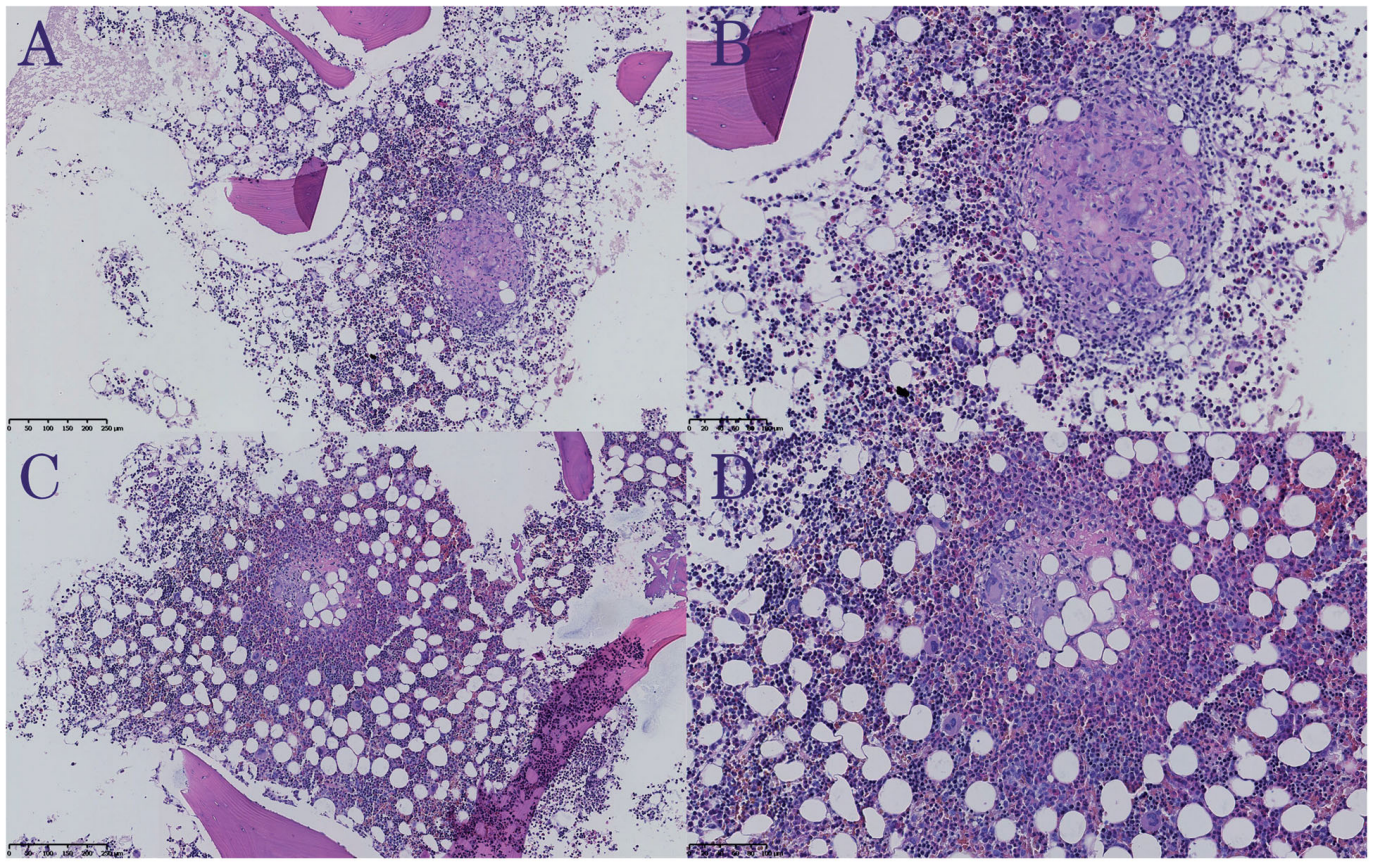
Pathological image of bone marrow biopsy. Pathological image showed necrotizing granuloma reactions in bone marrow (A and C: HE staining, 100×; B and D: HE staining, 200×), but acid-fast staining was negative.

A 32-year-old female patient was hospitalized due to "fever and headache for 20 days and dyspnoea for 10 days." The highest recorded body temperature reached 39.2 °C. The effect of anti-infection therapy with azithromycin and piperacillin-tazobactam given in the previous hospital was unsatisfactory. Two days before transferring to our hospital, the patient underwent non-invasive mechanical ventilation due to ARDS. According to her medical record, she became pregnant with twins after IVF-ET, which was given more than four months prior due to infertility caused by bilateral fallopian tube blockage. The results of the laboratory tests on admission were as follows: CBC: WBC (2.6 × 10^9^/L), neutrophil (1.0 × 10^9^/L), platelet (70 × 10^9^/L) and haemoglobin (95 g/L) counts; TB-IGRA: positive; serum biochemical indices: alanine aminotransferase (ALT, 151 U/L) and aspartate aminotransferase (AST, 141 U/L) levels; lumbar puncture:cerebrospinal fluid (CSF) pressure of 150 mmH_2_O; routine CSF analysis: nucleated cell (50 × 10^6^/L) and monocyte (70%) counts; and CSF biochemical analysis: protein (0.67 g/L), glucose (1.43 mmol/L) and chlorine (116 mmol/L) levels. A chest HRCT (Figure 1) showed multiple miliary nodular lesions distributed diffusely in both lungs, which were partially clustered and consolidated. A bone marrow biopsy (Figure 2) showed necrotizing granulomatous inflammation with positive TB-DNA detection results. Her diagnosis was as follows: (1) acute miliary TB; (2) tuberculous meningitis; (3) bone marrow TB; (4) ARDS; (5) abnormal liver function: liver TB? (6) diamniotic and dichorionic twin pregnancy; and (7_ after IVF-ET. The HL_2_EO regimen (isoniazid, rifapentine, ethambutol and levofloxacin combination) was given for TB treatment, along with anti-inflammatory therapy with prednisone (30 mg orally once a day). Her symptoms improved significantly, and she was discharged from our hospital 20 days later. The HL_2_EO regimen was given for subsequent TB treatment, and the dosage of prednisone was reduced to 25 mg and then reduced by 5 mg per week until it was stopped. Six months later, the patient had completely returned to normal, as evidenced by the obviously improved lesion shown on a chest CT scan (Figure 1).

## Discussion

Among 74 pregnant women with tuberculosis infections, 23 women had miliary tuberculosis. In pregnancy with tuberculosis, the incidence of miliary tuberculosis (41.38%) in IVF-ET patients was significantly higher than that in naturally pregnant patients (24.44%). Endocrine and immune disorders may be the causes of miliary TB during pregnancy. Hormonal changes during pregnancy, especially oestrogen and progesterone changes, inhibit the immune function of lymphocytes and reduce the resistance of the mother [9,10]. Glucocorticoids are often given to sensitize the ovaries to gonadotropin stimulation in IVF treatment [11]. Meanwhile, follicular development after ovulation induction can elevate the oestradiol level. Both glucocorticoids and oestradiol can suppress the immune system, thus making pregnant women vulnerable to TB infection or relapse and promoting the occurrence of miliary TB. In addition, increased microvascular permeability in pregnant women enables mycobacterium *M. tuberculosis* to enter the bloodstream, thus resulting in miliary TB. To date, more than 360 million people in China have been infected with M. tuberculosis [12], so pregnancies complicated by miliary TB are not rare and should be highly considered. In addition, pregnant women have an increased susceptibility to viral respiratory diseases. The most common respiratory virus to infect pregnant women is influenza [13], and COVID-19 is also common in pregnant women [14].

According to the retrospective analysis of an infertile population seeking assisted reproduction conducted by Singh et al. [15] in 2008, half of the patients had tubal factor infertility, and the prevalence of genital TB in tubal factor infertility reached 48.5%. Research by Parikh et al. [16] showed that the incidence rate of genital TB in women with tubal factor infertility was 39%. In our study, 13 patients reported having one or more abortions, and genital TB should not be excluded from the causes of miscarriage. IVF and ET techniques enable fertilized eggs to bypass damaged fallopian tubes and allow infertile patients to become pregnant [17], thus resulting in the coexistence of pregnancy and genital TB. In addition, pregnant women have an increased susceptibility to infection. In particular, women from countries with high TB incidence should be screened for active or latent TB and treated before IVF treatment [18].

Miliary TB lacks specific clinical characteristics, and typical miliary lesions may not be visible radiographically; hence, it is difficult to diagnose this disease. The common clinical symptoms in this study included fever, dyspnoea, cough, headache, abdominal pain and chest pain, basically consistent with those of pregnant women with miliary TB found in other studies [19]. HRCT is relatively more sensitive and shows randomly distributed miliary nodules, playing a significant role in the diagnosis of miliary TB. Fundus examination for choroid tubercles, histopathological examination of tissue biopsy specimens, and rapid culture methods for the isolation of *M. tuberculosis* in sputum, body fluids, and other body tissues aid in confirming the diagnosis [20,21]. According to our data, the disease is more likely to occur in the first trimester of pregnancy after IVF-ET and in the second trimester of natural pregnancy; in other words, the symptom onset in the former group is earlier than that in the latter group. The time from IVF-ET to symptom onset in our study was in line with those reported in other studies [22,23]. Our study shows that the time from symptom onset to diagnosis is relatively long, suggesting that it is difficult to diagnose the disease and that misdiagnosis is common in the early stages.

For the mother, miliary TB may cause multiple organ involvement, such as liver dysfunction and bone marrow TB, as well as ARDS [22], for which ECMO has proven to be an effective treatment [24]. The disease may also have a far-reaching impact on the foetus, such as a higher risk of miscarriage and preterm delivery, intrauterine growth retardation and stillbirth, and congenital TB [25,26]. Early diagnosis and effective treatment may avoid tuberculosis-related complications in both the mother and foetus [27]. In our study, extrapulmonary organ involvement occurred in ten patients; half of the patients developed respiratory failure, and some progressed to ARDS. A related study showed that advanced age, ARDS, consciousness disturbances, and high blood urea nitrogen levels are factors associated with poor prognosis in patients with miliary TB [28]. Patients usually have a favourable prognosis with prompt, effective respiratory support, and anti-TB and glucocorticoid therapies [29]. However, the effect of glucocorticoids as an adjuvant therapy has not been properly studied [30,31]. Factors associated with a good prognosis in our study included young age, no consciousness disturbances, and prompt, effective respiratory support and anti-TB treatment.

Because this study is a retrospective single-centre study, we could not obtain the number of pregnancies or the number of women undergoing IVF-ET in this area. It is impossible to calculate the incidence of miliary tuberculosis in pregnancy women and the incidence of miliary tuberculosis in women undergoing IVF-ET.

## Conclusions

Miliary tuberculosis can occur in pregnant patients, especially in pregnant patients after IVF-ET. Symptoms often appear in the first trimester of pregnancy after IVF-ET and in the second trimester of natural pregnancy. Fever and dyspnoea are common clinical symptoms of miliary TB. The occurrence of miliary TB should be considered when routine anti-infection effects are not satisfied. Multiple miliary nodules can be observed on a chest HRCT scan, and pulmonary consolidation may be detected at the disease progression stage in some patients. Pancytopenia is a CBC marker of pregnancy complicated with bone marrow TB. Half of these patients may develop respiratory failure, and some may progress to ARDS. Patients usually have a favourable prognosis with prompt, effective respiratory support and anti-TB therapy. Therefore, infertile patients from countries with high TB incidence should be required to undergo TB screening before undergoing IVF-ET, and preventive anti-TB treatment should be given to patients with latent TB infections or untreated TB disease.

## Data Availability

Access to data is regulated by Chinese law. Data are available from the Sichuan University Hospital for researchers who meet the criteria required by Chinese law for access to confidential data. The contact person will distribute data upon request to qualified researchers: Kaige Wang, Department of Pulmonary and Critical Care Medicine, West China Hospital, Sichuan University, 532951643@qq.com.
